# Thiamine Alleviates High-Concentrate-Diet-Induced Oxidative Stress, Apoptosis, and Protects the Rumen Epithelial Barrier Function in Goats

**DOI:** 10.3389/fvets.2021.663698

**Published:** 2021-05-20

**Authors:** Yi Ma, Ying Zhang, Mawda Elmhadi, Hao Zhang, Hongrong Wang

**Affiliations:** Laboratory of Metabolic Manipulation of Herbivorous Animal Nutrition, College of Animal Science and Technology, Yangzhou University, Yangzhou, China

**Keywords:** thiamine, goats, subacute rumen acidosis, apoptosis, oxidative stress, immune function, tight junction proteins

## Abstract

High-concentrate diets are continually used in ruminants to meet the needs of milk yield, which can lead to the occurrence of subacute rumen acidosis in ruminants. This study investigated the protective effects of dietary thiamine supplementation on the damage of the ruminal epithelium barrier function in goats fed a high-concentrate diet. Twenty-four healthy Boer goats (live weight of 35.62 ± 2.4 kg; age, 1 year) were randomly assigned into three treatments, with eight goats in each treatment, consuming one of three diets: a low-concentrate diet (CON; concentrate/forage, 30:70), a high-concentrate diet (HC; concentrate/forage, 70:30), or a high-concentrate diet with 200 mg of thiamine/kg of dry matter intake (HCT; concentrate/forage, 70:30) for 12 weeks. The additional dose of thiamine was based on our previous study wherein thiamine ameliorates inflammation. Compared with HC treatment, the HCT treatment had markedly higher concentrations of glutathione, superoxide dismutase, and glutathione peroxidase and total antioxidant capacity (*P* < 0.05) in plasma and rumen epithelium. The results showed that the apoptosis index was lower (*P* < 0.05) in the HCT treatment than in that of the HC treatment. Compared with the HC treatment, permeability and the electrophysiology parameter short circuit current for ruminal epithelial tissue were significantly decreased (*P* < 0.05) in the HCT treatment. The immunohistochemical results showed that the expression distribution of tight junctions including claudin-1, claudin-4, occludin, and zonula occludin-1 (ZO-1) was greater (*P* < 0.05) in the HCT treatments than in the HC treatment. The mRNA expression in the rumen epithelium of ZO-1, occludin, claudin-1, B-cell lymphoma/leukemia 2, nuclear factor erythroid-2 related factor 2 (Nrf2), superoxide dismutase 2 (SOD2), glutathione peroxidase 1, and the phase II metabolizing enzymes quinone oxidoreductase and heme oxygenase in the HCT group was significantly increased in comparison with the HC diet treatment (*P* < 0.05), whereas the mRNA expression of caspase 3, caspase 8, caspase 9, bcl-2 associated X protein, lipopolysaccharide binding protein, toll-like receptor 4, nuclear factor kappa-B (NFκB), tumor necrosis factor alpha, interleukin-1β, interleukin, and tumor necrosis factor receptor-associated factor 6 decreased significantly in the HCT treatment (*P* < 0.05). Compared with the HC treatment, the HCT diet significantly increased the protein expression of ZO-1, occludin, claudin-1, NQO1, HO-1, SOD2, serine/threonine kinase, p-Akt, Nrf2, and p-Nrf2; conversely, the expression of NFκB-related proteins p65 and pp65 was significantly decreased (*P* < 0.05). In addition, thiamine relieved the damage on the ruminal epithelium caused by the HC diet. The results show that dietary thiamine supplementation improves the rumen epithelial barrier function by regulating Nrf2–NFκB signaling pathways during high-concentrate-diet feeding.

## Introduction

In the intensive ruminant production industry, high-concentrate diets are used to feed goats or dairy to meet the global meat and milk demand (1). Although the richness in fermentable carbohydrate diets can improve ruminant production efficiency in the short term, it can also cause subacute rumen acidosis (SARA), a common metabolic disorder, when administered for long periods (2). The feeding of excessive high-concentrate diets caused an increase in short-chain fatty acids and lactate and resulted in a high reduction in ruminal pH (3, 4). This decline in ruminal pH can enhance the lysis of gram-negative bacteria and release lipopolysaccharide (LPS) that translocate into the bloodstream, which results in damage of the ruminal epithelium (5). Several studies have demonstrated that low pH can affect the integrity and permeability of the rumen epithelium (6–8). Moreover, the negative changes in barrier function of the rumen epithelium facilitate the translocation of LPS, spreading it into the circulatory system and resulting in systemic inflammation (9). Oxidative stress is related to the pathogenesis of many pathological conditions, including sepsis, mastitis, enteritis, and pneumonia (10). Therefore, it is a novel thought to consider the relationship between inflammation and oxidative stress in ruminal epithelial barrier function protection.

The tight junctions (TJs) are composed of transmembrane proteins that play a crucial role in regulating the permeability of the epithelial barrier and mediate adhesive function (11, 12), including the transmembrane proteins claudins and the adaptor proteins ZO-1, zonula occludin-2 (ZO-2), and zonula occludin-3 (ZO-3) (13–15). Previous studies have determined that the variation of TJ protein expression and distribution is the dominant reason for the disruption of the epithelial TJ barrier (16, 17), whereas the impairment of TJs causes a “leaky” epithelial barrier and then invasion of susceptible LPS, resulting in inflammatory responses (18). Occludin and ZO-1 are relevant to various junctional functions (19). The change of expression for occludin and ZO-1 induces an increase in transepithelial electrical resistance (20), resulting in an increase in paracellular permeability (21).

The damage in the epithelial barrier is closely linked with the trigger of inflammation and oxidative stress. Previous studies have demonstrated that excess feeding of a high-concentrate diet intensifies the activation of NFκB *via* LPS/TLR4 signaling (4, 22). Meanwhile, the transcription factor NFκB can be activated by oxidative stress and causes cytotoxicity and recruitment of apoptotic machinery (23–25). The suppression of the antioxidant system was caused by the high-grain diet through the influx of LPS concentration in peripheral blood (26). The gene Nrf2 takes a significant role in regulating the activation of the defense system, which leads to the protection of cells against the adverse impacts of oxidative stress and suppresses apoptosis (27). Accumulation of LPS causes the lower expression of Nrf2, resulting in suppression of the antioxidant system (28). The coordination between Nrf2 and NFκB is indispensable to enhance body health (29).

Thiamine is also known as vitamin B_1_ and plays a crucial role in carbohydrate metabolism by participating in the pentose phosphate pathway and tricarboxylic acid cycle. Thiamine also increases antioxidant formation and NADPH levels (30). Some researchers showed that thiamine might act as a potent antioxidant by scavenging free radicals to apoptotic inhibition (31). Simultaneously, exogenous thiamine supplementation reduced inflammation in ruminal epithelium during high-concentrate feeding *via* the suppression of NFκB activation (4, 32). A careful review of literature revealed that there are no data on the protective effect of thiamine on the rumen epithelium oxidative damage induced by a high-concentrate diet. Therefore, this present study aimed to investigate whether thiamine feeding increases TJ expression in rumen epithelium by modulating the Nrf2–NF-κB pathways, thereby protecting the rumen epithelial barrier function.

## Materials and Methods

All trial plans and methods have been authorized by the Animal Ethics Committee of Yangzhou University, China. The study was conducted under the Care and Use of Laboratory Animals guidelines (SXXY 2015-0054).

### Animals, Diet, and Experimental Design

There were 24 healthy Boer goats with no clinical sign, averaging 35.62 ± 2.4 kg in body weight (BW), at the beginning of the trial. The goats were randomly assigned into three treatments, with eight goats in each treatment, consuming one of three diets: (1) low-concentrate diet (CON; concentrate/forage, 30:70), (2) high-concentrate diet (HC; concentrate/forage, 70:30), and (3) high-concentrate diet with 200 mg of thiamine/kg of dry matter intake (DMI) (HCT; concentrate/forage, 70:30) for 12 weeks. The dietary composition is presented in Table 1. The added dose of thiamine was based on our previous study wherein thiamine ameliorates inflammation (4).

**Table 1:** Ingredient and nutrient composition of the experimental diets.

		**Dietary treatment**	
**Item**	**CON**	**HC**	**HCT**
Ingredient (% of DM)			
Corn grain	14.54	60.23	60.23
Soybean meal	12.50	7.10	7.10
Oat grass hay	56	24	24
Alfalfa hay	14	6	6
Calcium hydrophosphate	1.38	0.52	0.52
Limestone	0.58	1.15	1.15
Salt	0.50	0.50	0.50
Premix^a^	0.50	0.50	0.50
Nutrient composition			
DE (MJ/kg)	15.2	15.4	15.4
CP (%)	17.88	17.75	17.75
NDF (%)	43.68	27.53	26.53
ADF (%)	25.32	15.15	15.15
Starch (%)	26.17	51.56	51.56
Calcium (%)	1.46	1.32	1.32
Phosphorus (%)	0.54	0.57	0.57
Thiamine (mg/kg of DM)	1.56	1.82	204.82

*CON, control; HC, high-concentrate diet; HCT, high-concentrate diet supplemented with 200 mg of thiamine/kg of dry matter intake*.

a *The premix consisted of the following ingredients per kilogram of diet: 6.00 × 10^3^ IU of vitamin A, 3.0 × 10^3^ IU of vitamin D, 82.0 mg of vitamin E, 6.15 mg of Cu, 70.0 mg of Fe, 65.0 mg of Zn, 47.0 mg of Mn, 0.135 mg of I, 0.115 mg of Co, and 0.115 mg of Mo*.

The goats were housed in small individual pens. The goats were fed at 0700 h and 1,800 h, at one-half of the allowed amount daily at each feeding, and had free access to freshwater during the trial period. Thiamine was mixed with the concentrate to be licked by the goats. The feed ingestion and physical condition of the animals were observed daily. All goats were slaughtered at the end of the trial, at 3 h following the final feeding.

### Sample Collection and Analysis

After the goats were slaughtered, a representative sample of ~50 ml rumen fluid was collected to calculate the pH value instantly with a pH meter (Sartorius, Goettingen, Germany). The ruminal fluid was filtered through a four-layer cheesecloth, centrifuged at 10,000 × *g* for 15 min at 4°C, and stored at −20°C for later analysis. Blood samples were collected from the jugular vein using 5-ml vacuum tubes with sodium heparin (Lab Anim Tech Develp Co., Ltd., Beijing, China). Plasma was obtained *via* centrifugation at 3,000 × *g* at 4°C for 15 min and then stored at −20°C until further analysis. Within 5 min of slaughter, representative ventral epithelial tissues were exteriorized from the rumen and washed three times in precooled phosphate-buffered saline. A part of the sample (~50 cm^2^) was used for the Ussing chamber measurement. Approximately 10 g epithelium tissue was dissected into smaller pieces and transferred into liquid nitrogen for protein and RNA analysis. For histological analysis, the whole ventral sac rumen tissues were also fixed in either 4% paraformaldehyde or 2.5% glutaraldehyde (Sigma, St. Louis, MO) for histomorphometric microscopic analysis.

### Measurements of Physiological Parameters in Rumen Fluid Analysis

Free LPS in the preprocessed rumen fluid was measured (33) by a chromogenic end-point Tachypleus amebocyte lysate assay kit (Rongbai Biological Technology Co., Ltd., Shanghai, China) with a minimum detection limit value of 0.01 endotoxin units (EU)/ml. Briefly, the rumen fluid was centrifuged at 10,000 × *g* for 45 min, and then the collected rumen fluid was diluted 100,000-fold for measurement of LPS. The detailed determination methods refer to Dai's previous studies (34). The thiamine concentration in ruminal fluid was tested with a micro method ELISA kit (Xin Yu Biotech Co., Ltd. Shanghai, China) following the manufacturer's explanatory memorandum. For the determination of volatile fatty acid (VFA), the rumen fluid mixtures were centrifuged at 3,000 *g* for 15 min, and then 25% (w/v) metaphosphoric acid was added into the collected supernatants. The VFA concentration was measured by gas chromatography (GC-14B, Shimadzu, Kyoto, Japan; Qin, 1982).

### The Concentration Analyses of Thiamine, LPS, LBP, Cytokine, and IgA in the Blood

Thiamine and LPS in plasma were determined using similar methods as described above for rumen fluid and a lower measurement limit of LPS as 0.1 endotoxin units/ml. The LBP concentrations were performed using an ELISA kit test (Chinese Horseshoe Crab Reagent Manufactory, Xiamen, China) with a detection range of 15.6–1,000 ng/ml. The determination of IgA in serum was *via* an immunoturbidimetry assay kit (Bethyl Lab., Inc., Montgomery, USA). The cytokine in plasma was determined using the following commercial kits according to the manufacturer's instructions: TNF-α (Bio Source/Med Probe, Camarillo, CA), IL-1β (MyBioSource Inc., San Diego, CA), IL6 (MyBioSource Inc., San Diego, CA), and IL10 (Bethyl Lab., Inc., Montgomery, USA). The absorbance of all samples was read at 450 nm using a microplate reader (BioTek Instruments, Winooski, VT). The concentrations of TNF-α, IL-1β, IL6, and IL-10 had a detection range of 15.6–500 ng/ml, 31.25–2,000 pg/ml, 15.6–500 ng/ml, and 5–1,000 pg/ml, respectively, and the variation coefficients for inter- and intra-assay were no more than 10%.

### Oxidative Enzyme Activities in Rumen Epithelial Tissue Measurement

The oxidative enzyme activities in rumen epithelial tissue were measured using commercial kits (Jiehuigao Biological Technology Co., Ltd., Beijing, China) according to the manufacturer's test specification for the following indicators: malondialdehyde (MDA), GSH and oxidized glutathione, glutathione reductase, GSH-Px, SOD, and T-AOC. All estimated values were standardized to the concentration of the total protein. BCA Protein Assay Kit (Beyotime Biotechnology Institute, Shanghai, China) was used to assess the protein concentrations.

### Oxidative Enzyme Activities in Plasma Measurement

The oxidative enzyme activities in plasma were determined using the same assay kit described above for rumen epithelial tissue according to the manufacturer's instructions. The absorbance of all samples was read at 550 nm using a microplate reader (BioTek Instruments, Winooski, VT). The coefficient of variation was <2%.

### Ussing Chamber Measurements in Ruminal Epithelial Sample

According to Klevenhusen's methods (8), the Ussing chamber technique was used to measure electrophysiological properties and the permeability of rumen epithelium tissue. Briefly, an ~50-cm^2^ ruminal epithelium tissue in ventral sac was isolated, the ventral rumen epithelium tissues was cleaned by immersion in buffer solution firstly, and then the epithelium was peeled away from the muscle layer. The isolated epithelium tissues were picked to pieces of about 2 cm^2^ and mounted between the two halves of the incubation chambers. Both halves of the chambers were instantly filled with buffer solution (8) and were gassed with carbogen gas (95% O_2_ and 5% CO_2_). Buffer temperature was maintained stationary at 39°C during the measurement process. Fluorescein 5(6)-isothiocyanate (FITC) and horseradish peroxidase (HRP) (Sigma-Aldrich, Schnelldorf, Austria) as markers were used to evaluate permeability. FITC and HRP were added to the mucosal side of each chamber, and hourly samples were taken from the serosal side to measure the flux through the epithelium. The electrophysiological parameters for Isc, tissue conductance (Gt), and transmembrane potential difference (PD) were determined by the Ussing chamber system and Acquire and Analyze 2.3 software.

### Histological and Microscopic Analysis

Exemplars of the rumen wall were fixed *via* 4% paraformaldehyde solution and then embedded in paraffin, sectioned, and dyed by hematoxylin and eosin (H&E) for histological observation. A scoring criterion was adopted for determining histological damage as described previously (35). Briefly, the damage score was translated into three levels from 0 to 3, including superficial epithelial injury, moderate including focal erosions, and severe including multifocal erosions. Microscopic analysis *via* scanning electron microscopy (SEM) was adapted from Liu et al. (36). Briefly, the washed rumen tissue was immediately fixed in 2.5% glutaraldehyde for 24 h, transferred to 1% osmium for 1 h, then dehydrated *via* ethanol solutions, and coated with gold after having been kept under critical-point drying. The sample went through a series of treatments, cut into ultrathin sections (70–90 nm), and observed using transmission electron microscopy (Hitachi H-7650, Hitachi Technologies, Tokyo, Japan).

### TUNEL Analysis

Apoptotic epithelial cells in rumen epithelium tissue were analyzed using TUNEL staining kits (Abcam, Shanghai, China) according to manufacturer's test specification. In TUNEL staining, the nucleotides attached by TdT were tagged either directly with a fluorescent label or with a chemical label that can be indirectly linked to either a fluorescent label or an enzyme. TUNEL-positive nuclei were dyed as brown-stained nuclei, which stated the existence of DNA fragmentation as a result of apoptosis. The positive rate was determined in randomly selected fields adapted from Tao's method (37).

### Immunohistochemistry of Ruminal Epithelium Tight Junction Protein Analysis

The expression and distribution of ruminal epithelium tight junction proteins were performed *via* immunohistochemistry. The following antibodies were used in the immunohistochemistry analysis: claudin-1 (ab211737; Abcam), claudin-4 (ab210796; Abcam), occludin (ab167161, Abcam), and ZO-1 (ab214228, Abcam). Specimens were embedded in paraffin after having been fixed *via* 4% paraformaldehyde solution, sectioned and incubated with antibodies, and dyed by hematein for light microscope observation. Image Pro Plus v.6.0 software was used to choose the same brown color as the consistent criterion for estimating all photos. Each photo was determined to acquire the cumulative optical density of each image.

### Total RNA Extraction and Real-Time PCR Analysis

Total RNA was extracted from 100 mg of rumen epithelium tissue *via* Tissue Total RNA Isolation Kit V2 (Vazyme Biotech Co., Ltd. Nanjing, China) according to the manufacturer's instructions. RNA integrity was quantified on A260/A280 ratio by Nano Drop 2000 (Thermo Fisher Scientific, Inc., Waltham, MA, USA). Only those samples with ratio A260 to A280 between 1.8 and 2.1 were subsequently adopted for cDNA trials. Then, cDNA synthesis was performed using 500 ng of the total RNA template by HiScript III RT SuperMix for qPCR (+gDNA wiper) (Vazyme Biotech Co., Ltd). Target primers were designed using Primer Premier Software 5.0 and synthesized by Sangon Biotech (Shanghai, China). The primer details are presented in Table 2. Real-time PCR was performed using ChamQTM SYBR® qPCR Master Mix Kit (Vazyme, Nanjing, China) with 7500 Fast Real-Time PCR System (Applied Bio-systems, CA, USA) according to the manufacturer's instructions. All processes were measured in triplicate. The quantitative PCR results were analyzed using the 2^−ΔΔCt^ method (38). GAPDH gene expression was constant and was the only internal control gene in all samples.

**Table 2:** Primers for quantitative real-time PCR.

**Gene name**	**Sequences (5′-3′)**	**GenBank accession**
LBP	F: CGGATGACATGATTCCGCCT	XM_005688550.3
	R: GAGCACAAAGGCCTCGATCT	
TNF-α	F: CAAGTAACAAGCCGGTAGCC	XM_005696606.3
	R: AGATGAGGTAAAGCCCGTCA	
TLR-4	F: GCAGCCATAACTTCTCCAGGTTCC	NM_001285574.1
	R: TCTCCACGGCCACCAGCTTC	
IL-6	F: ACACTGACATGCTGGAGAAGATGC	NM_001285640.1
	R: CCGAATAGCTCTCAGGCTGAACTG	
IL-1β	F: CATGTGTGCTGAAGGCTCTC	XM_013967700.2
	R: AGTGTCGGCGTATCACCTTT	
NF-KB	F: TGGCGAGAGGAGCACAGACAC	XM_018043384.1
	R: TGACCAGCGAGATGCGGACTG	
TRAF-6	F: ACGACGTGGAATTTGACCCA	XM_018059227.1
	R: CTTCCCGCAAAGCCATCAAG	
Nrf2	F: ATCCAGATGCTCACCATGCG	XM_005674733.2
	R: CCCAATGCAGGACTTGGTCT	
NQO1	F: CAACAGACCAGCCAATCA	XM_005692193.3
	R: ACCTCCCATCCTTTCCTC	
HO-1	F: GAACGCAACAAGGAGAAC	NM_001285567.1
	R: CTGGAGTCGCTGAACATAG	
SOD2	F: GCTTGCAGATTGCTGCTTGT	XM_018053428.1
	R: TGGCCTTCAGATAATCGGGC	
GPx1	F: CCTGAAGTACGTCCGACCAG	XM_005695962.3
	R: GCAGAGTCTCCCGAAGGAAG	
Caspase 3	F: AGCAAACCTCAGGGAAAC	XM_018041755.1
	R: CTTAGAAGCACGCAAATAA	
Caspase 8	F: GGCTCCTCTGAGATGCTG	XM_018060934.1
	R: TGCTCCCGTGCTATGCTAT	
Caspase 9	F: TCCTTTGTTCATCTCCTGCTTG	XM_005690814.3
	R: TTTTCCTTGGCTTGGCTTTG	
Bcl-2	F: AGGCTCACAGCACACTCTTC	XM_018039337.1
	R: GGCCTGTGGGCTTCACTTAT	
Bax	F: TGAAGCGCATTGGAGATG	XM_013971446.2
	R: GGCCTTGAGCACCAGTTT	
ZO-1	F: TGGCAATGGTTAATGGCGTCTCC	XM_018066118.1
	R: TGCCTCCTCGTCGTAACTGTCC	
Occludin	F: GCCTGTGTTGCCTCCACTCTTG	XM_018065677.1
	R: CATAGCCATAGCCACTTCCGTAGC	
Claudin-1	F: GCTGTGGATGTCGTGCGTGTC	XM_005675123.3
	R: TGCCTCCTCGTCGTAACTGTCC	
Claudin-4	F: TCATCGGCAGCAACATCGTCAC	XM_005697785.2
	R: CAGCAGCGAGTCGTACACCTTG	
GAPDH	F: GGGTCATCATCTCTGCACCT	XM_005680968.3
	R: GGTCATAAGTCCCTCCACGA	

*LBP, lipopolysaccharide binding protein; TNF-α, tumor necrosis factor alpha; TLR 4, Toll-like receptor 4; TRAF-6, TNF receptor-associated factor 6; Nrf2, nuclear factor erythroid-2 related factor 2; HO-1, heme oxygenase-1; NQO1, quinone oxidoreductase 1; SOD2, superoxide dismutase 2; GPx1, glutathione peroxidase 1; Bax, Bcl-2 associated X protein; Bcl-2, B-cell lymphoma/leukemia 2; ZO-1, zonula occludens-1; F, forward; R, reverse*.

### Western Blotting Analysis

The total protein was fetched from the ruminal epithelium in all samples by mercantile kit (Thermofisher Scientific, New York, USA) based on the manufacturer's protocol. After that, the concentration of protein was measured *via* a special protein assay kit (Beyotime Biotechnology, Jiangsu, China). In this study, information on primary antibodies is shown in the following: anti-ZO-1 (diluted at 1:1,000, Abcam), anti-occludin (diluted at 1:1,000, Abcam), anti-claudin-1 (diluted at 1:1,000, Abcam), anti-catalase (anti-CAT, diluted at 1:2,000, Protein Tech), anti-heme oxygenase-1 (anti-HO-1, diluted at 1:1,000, Abcam), anti-superoxide dismutase 2 (anti-SOD2, diluted at 1:5,000, Novus), anti-p65 (diluted at 1:300, Cell Signaling), anti-pp65 (diluted at 1:300, Cell Signaling), anti-Akt (diluted at 1:2,000, Santa Cruz Biotechnology), anti-pAkt (diluted at 1:2,000, Santa Cruz Biotechnology), anti-nuclear factor erythroid-2 related factor 2 (anti-Nrf2, diluted at 1:2,000, Protein Tech), anti-pNrf2 (diluted at 1:2,000, Protein Tech), and anti-β-actin (diluted at 1:1,500, Santa Cruz). Thereafter, the protein was incubated for 45 min *via* the secondary antibody horseradish peroxidase (HRP-conjugated goat anti-rabbit IgG, 1:1,000, Beyotime), and then signals were visualized using the enhanced chemiluminescence kit (Thermofisher Scientific, USA) followed by a Bio-Rad imaging detection system. The data were analyzed *via* Image J software. All gray values were quantified to β-actin and were expressed relative to the control (39). Each trial was repeated six times.

### Statistical Analysis

Data were analyzed using SPSS 21.0 software (SPSS Inc., Chicago, IL). The sex effect was involved in the original statistical model, giving no significant result (*P* > 0.05). Thus, the sex effect was eliminated from the final model in which treatment was only the fixed effect. Statistical significance was assessed using one-way analysis of variance and Tukey's *post hoc* test between different treatments. Data were deemed statistically significant when *P* < 0.05.

## Results

### Ruminal pH, Metabolite Content, Cytokine, and IgA

As shown in Table 3, Boer goat had a markedly decreased ruminal pH value in the HC treatment compared with the HCT treatment (*p* < 0.05). The HC diet feeding induced the increase of free LPS and the decrease of thiamine in rumen fluid and plasma, while thiamine supplementation reversed the results, and the same trend as LPS has happened to LPB in plasma (*p* < 0.05). Furthermore, the contents of butyrate, propionate, and total VFA were significantly lower, whereas the acetate content was significantly higher in the HCT treatment than in the HC treatment (*p* < 0.05).

**Table 3:** Effects of thiamine supplementation on metabolites in the rumen and contents of thiamine, lipopolysaccharide, cytokine, and IgA in the blood of goats fed with a high-concentrate diet.

	**Dietary treatment**				
**Item**	**CON**	**HC**	**HCT**	**SEM**	***P*-value**
**RUMINAL**					
pH	6.15^a^	5.36^b^	6.05^a^	0.13	0.001
Thiamine (μg/L)	8.03^a^	2.59^c^	5.40^b^	0.21	0.004
LPS (× 10^3^ EU/ml)	26.46^a^	48.37^b^	31.65^a^	2.95	0.008
Acetate (mM)	42.08^a^	33.82^b^	46.32^a^	3.13	0.003
Propionate (mM)	14.62^c^	34.98^a^	19.28^b^	2.26	0.006
Butyrate (mM)	10.89^c^	17.64^a^	13.21^b^	0.54	0.003
TVFA (mmol/L)	67.64^c^	86.82^a^	78.88^b^	3.16	0.008
**BLOOD**					
Thiamine (μg/L)	15.67^a^	10.18^c^	12.64^b^	0.33	0.005
LPS (EU/ml)	0.28^b^	0.69^a^	0.35^b^	0.11	0.004
LBP (μg/ml)	26.49^b^	58.34^a^	34.54^b^	0.24	0.007
IgA (ng/ml)	61.34^a^	47.36^b^	58.69^a^	3.34	0.009
TNF-α (ng/L)	38.26	46.38	36.59	6.52	0.473
IL-1β (ng/L)	139.54^b^	216.39^a^	151.62^b^	13.79	0.008
IL6 (ng/L)	39.27^b^	65.82^a^	43.53^b^	5.56	0.017
IL10 (ng/L)	46.49	52.56	45.74	7.64	0.265

*Within a row, means without a common superscript letter differ (P < 0.05)*.

*TVFA, total volatile fatty acid; LBP, lipopolysaccharide binding protein; IgA, immune globulin; EU, endotoxin unit; IU, international unit; CON, control; HC, high-concentrate diet; HCT, high-concentrate diet supplemented with 200 mg of thiamine/kg of dry matter intake (n = 8 goats/treatment)*.

Goats fed with the HCT treatment had lower concentrations of cytokines TNF-α, IL-1β, IL6, and IL10 in blood than those in the HC treatment (*p* < 0.05). Moreover, the IgA concentration in the HCT treatment was significantly higher than in the HC treatment (*p* < 0.05).

### Morphological and Ultrastructural Analysis of Rumen Papillae

**Figure 1:**
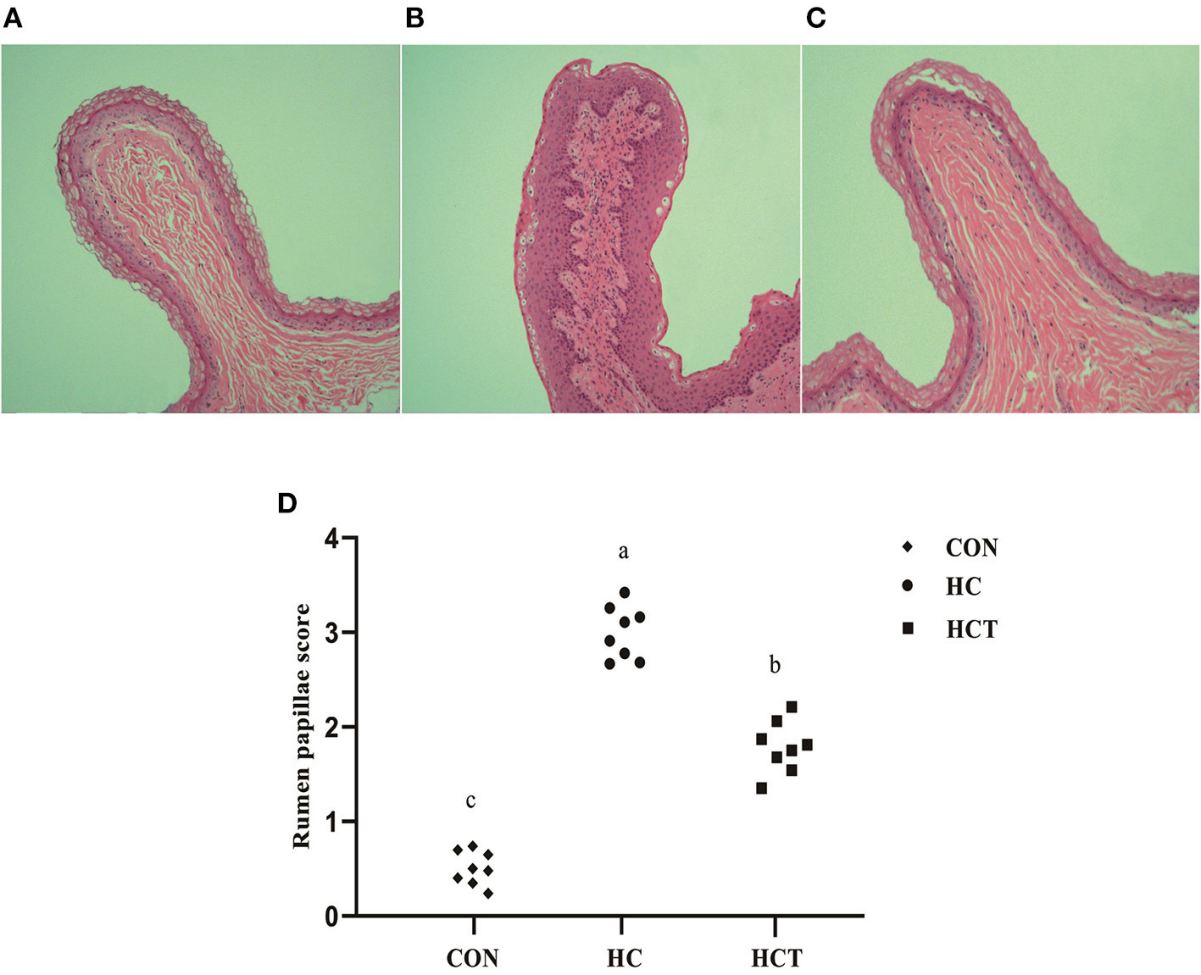
Representative histology sections of the rumen papillae of goats from the low-concentrate diet (CON), high-concentrate diet (HC), and high-concentrate diet with thiamine (HCT) treatment. Representative photomicrographs with H&E staining are shown. **(A)** The rumen papillae in the CON group were intact and showed no disruption. **(B)** In the HC treatment, the stratum corneum of the rumen papillae was severely damaged. **(C)** The rumen papillae showed a slight damage in the HCT treatment. **(D)** Rumen papillae injury score. All sections were stained with H&E and measured at ×400 magnification (*n* = 8 goats/treatment). The mean values in the figure without a common superscript letter differ (*P* < 0.05).

Representative light micrographs of rumen papillae cross-sections are shown in Figure 1. The result showed that the rumen papillae of the CON and HCT treatments remained intact, whereas the stratum corneum of the epithelium papillae was injured seriously in the HC treatment. Meanwhile, the histological damage score was significantly higher in the HC treatment compared with the CON and HCT treatment (*p* < 0.05).

**Figure 2:**
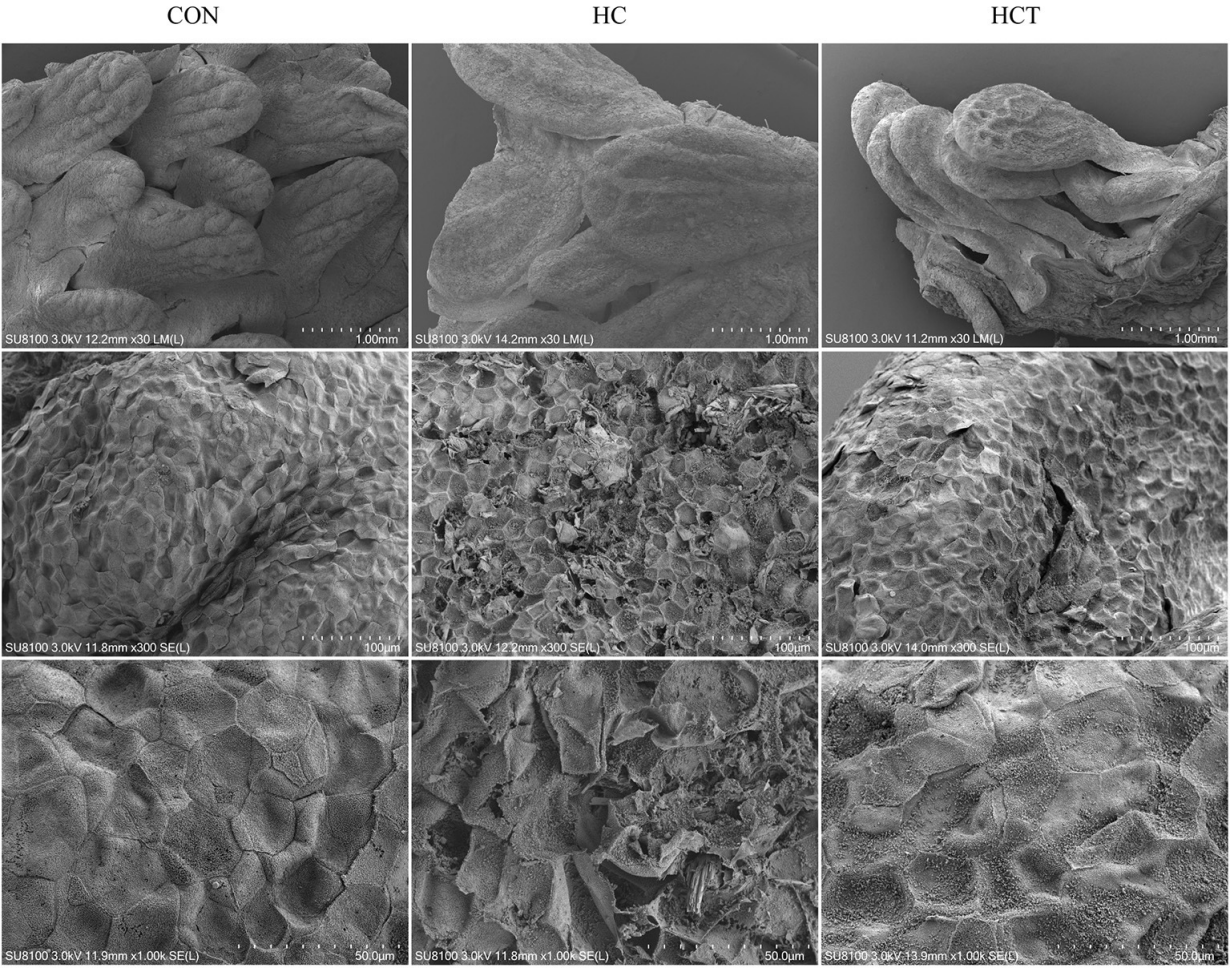
Comparisons of rumen papillae ultrastructure from the low-concentrate diet (CON), high-concentrate diet (HC), and high-concentrate diet with thiamine (HCT) treatments. Scanning electron microscopy (SEM) of the papillae surface in the three treatments with different scale bars (scale bar = 1.00 mm, 100 μm, and 50 μm, respectively).

Scanning electron micrographs of rumen papillae demonstrated that the HC diet feeding caused desquamation, indentations, and dead keratinized cells throughout the surface of the papillae, where profound cellular injury also appeared. Nevertheless, the CON and the HCT treatments showed normal histological morphology (Figure 2).

### Ussing Chamber Experiment

**Figure 3:**
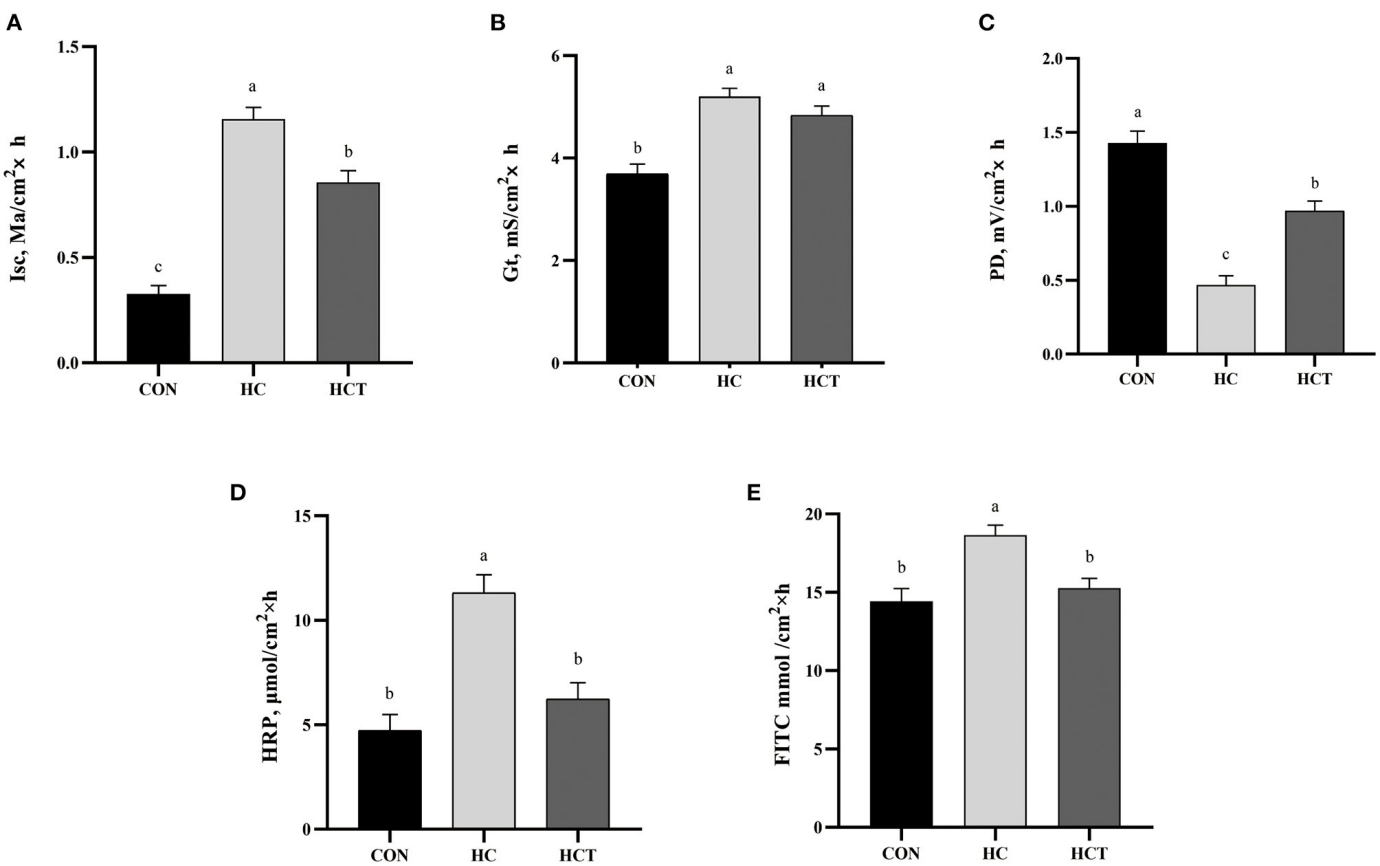
Effects of dietary thiamine supplementation on the electrophysiological properties: **(A)** short-circuit current (Isc), **(B)** tissue conductance (Gt), **(C)** transmembrane potential difference and tissue permeability measured as flux rate of **(D)** horseradish peroxidase and **(E)** fluorescein 5(6)-isothiocyanate. CON, low-concentrate diet; HC, high-concentrate diet; HCT, high-concentrate diet supplemented with 200 mg of thiamine/kg of dry matter intake (*n* = 8 goats/treatment). The mean values in columns without a common superscript letter differ (*P* < 0.05).

The HC diet treatment showed a significant up-regulation in Isc and Gt value and a significant down-regulation in PD values, whereas the HCT had a significant decrease in Isc and a significant increase in PD than the treatment only feeding a high-concentrate diet (*p* < 0.05; Figure 3). There remained no significant difference for Gt between the HC and HCT treatments. For the changing trend of permeability in ruminal epithelium, the HRP and FITC were found to be significantly lower in the HCT treatment than that in the HC treatment (*p* < 0.05), while their value was similar in the HCT and the CON treatments.

### Apoptotic Index

**Figure 4:**
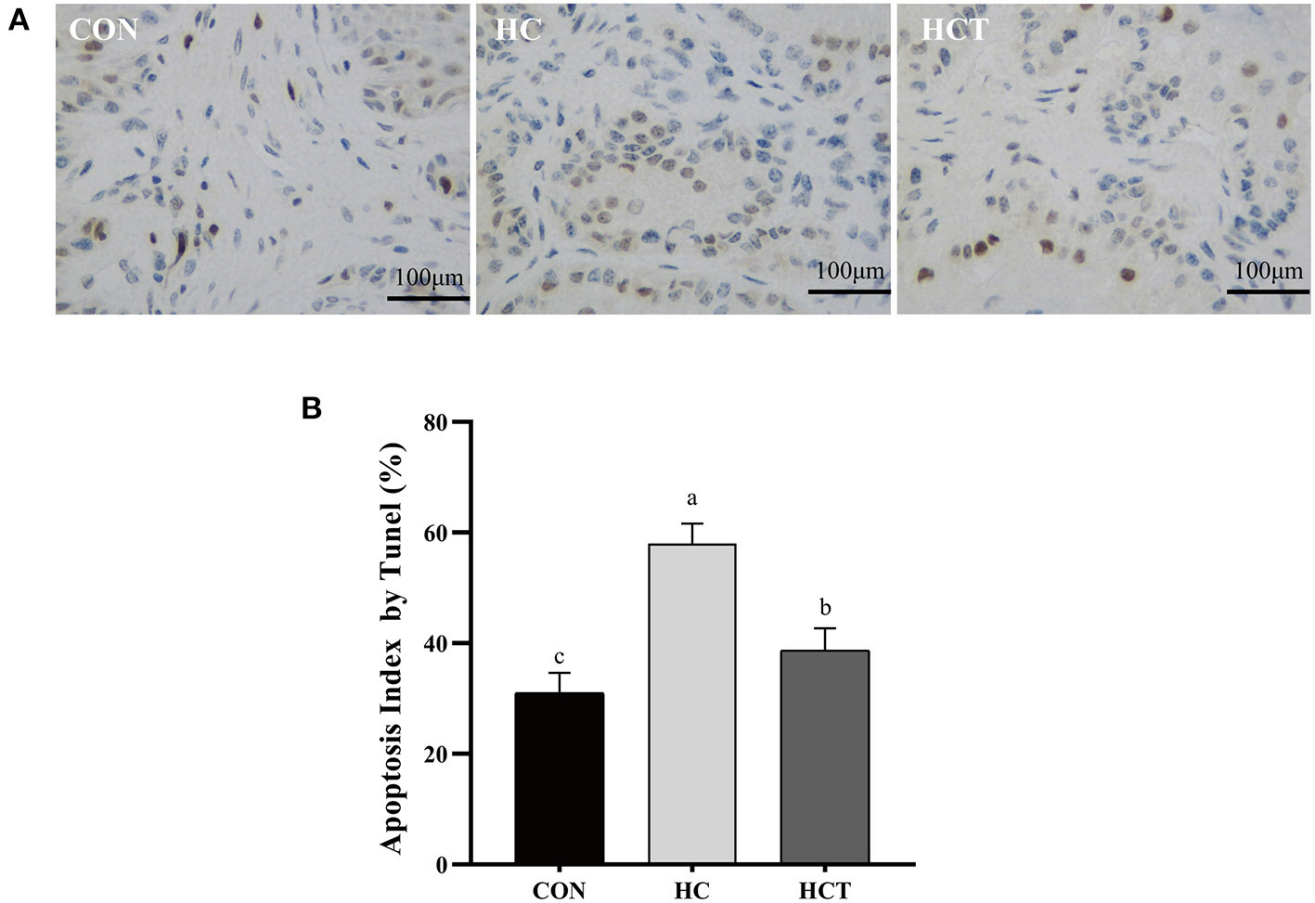
TUNEL comparison of the ruminal epithelium from the low-concentrate diet (CON), high-concentrate diet (HC), and high-concentrate diet with thiamine (HCT) treatments. Ruminal epithelium samples from each treatment were processed for evaluation of TUNEL-positive apoptotic cells. **(A)** Representative sections of rumen from the different treatments (CON, HC, and HCT treatments). **(B)** Apoptosis index analysis. All scale bars represent 25 μm (*n* = 8 goats/treatment). The mean values in columns without a common superscript letter differ (*P* < 0.05).

As shown in Figure 4, the apoptotic percentage in the HC treatment was greater than that of the CON treatment (*p* < 0.05). In contrast, the apoptotic percentage was significantly down-regulated after feeding an HCT diet (*p* < 0.05).

### Oxidative Stress Status

The plasma MDA enzyme activity was significantly higher, whereas GSH, GSH-PX, SOD, and T-AOC enzyme activities were significantly lower in the HC treatment than in the CON treatment (*p* < 0.05; Table 4), while there was no significant variation between the HC and the HCT treatments (*p* < 0.05). These indicators remained at a trend nearly similar to that observed in the plasma.

**Table 4:** Effects of thiamine supplementation on concentration of oxidative status index of plasma and rumen epithelium in goats with high concentrate diet.

**Item**	**Diet**				
	**CON**	**HC**	**HCT**	**SEM**	***P*-value**
**PLASMA**					
MDA (nmol/ml)	8.48^b^	11.67^a^	8.96^b^	1.02	0.036
GSH (U/ml)	13.69^a^	10.22^b^	12.87^a^	0.06	0.026
GSSG (U/ml)	3.73	4.06	3.84	0.07	0.322
GR (U/ml)	10.46	10.93	10.18	0.02	0.254
GSH-PX (U/ml)	436.54^a^	386.57^b^	418.66^a^	5.52	0.021
SOD (U/ml)	67.27^a^	38.49^c^	52.69^b^	2.63	0.004
CAT (U/ml)	6.24	5.48	5.86	0.09	0.068
T-AOC (U/ml)	13.68^a^	4.34^c^	8.76^b^	0.45	0.006
**RUMEN EPITHELIUM**					
MDA (nmol/mg protein)	0.74^b^	1.03^a^	0.78^b^	0.06	0.002
GSH (nmol/mg protein)	4.85^a^	3.02^b^	4.49^a^	0.31	0.008
GSSG (nmol/mg protein)	0.21	0.24	0.22	0.03	0.276
GR (U/g protein)	4.28	4.39	4.13	0.26	0.244
GSH-PX (U/mg protein)	54.47^a^	38.65^b^	57.85^a^	7.24	0.002
SOD (U/mg protein)	166.72^a^	149.62^b^	163.43^a^	6.57	0.006
T-AOC (U/ mg protein)	0.93^a^	0.43^c^	0.68^b^	0.09	0.007

*Within a row, means without a common superscript letter differ (P < 0.05)*.

*MDA, malondialdehyde; GSH, reduced glutathione; GSSG, oxidized glutathione; GR, glutathione reductase; GSH-Px, glutathione peroxidase; SOD, superoxide dismutase; CAT, catalase; T-AOC, total antioxidant capacity; CON, control; HC, high-concentrate diet; HCT, high-concentrate diet supplemented with 200 mg of thiamine/kg of dry matter intake (n = 8 goats/treatment)*.

### Immunohistochemistry of Ruminal Epithelium Tight Junction

**Figure 5:**
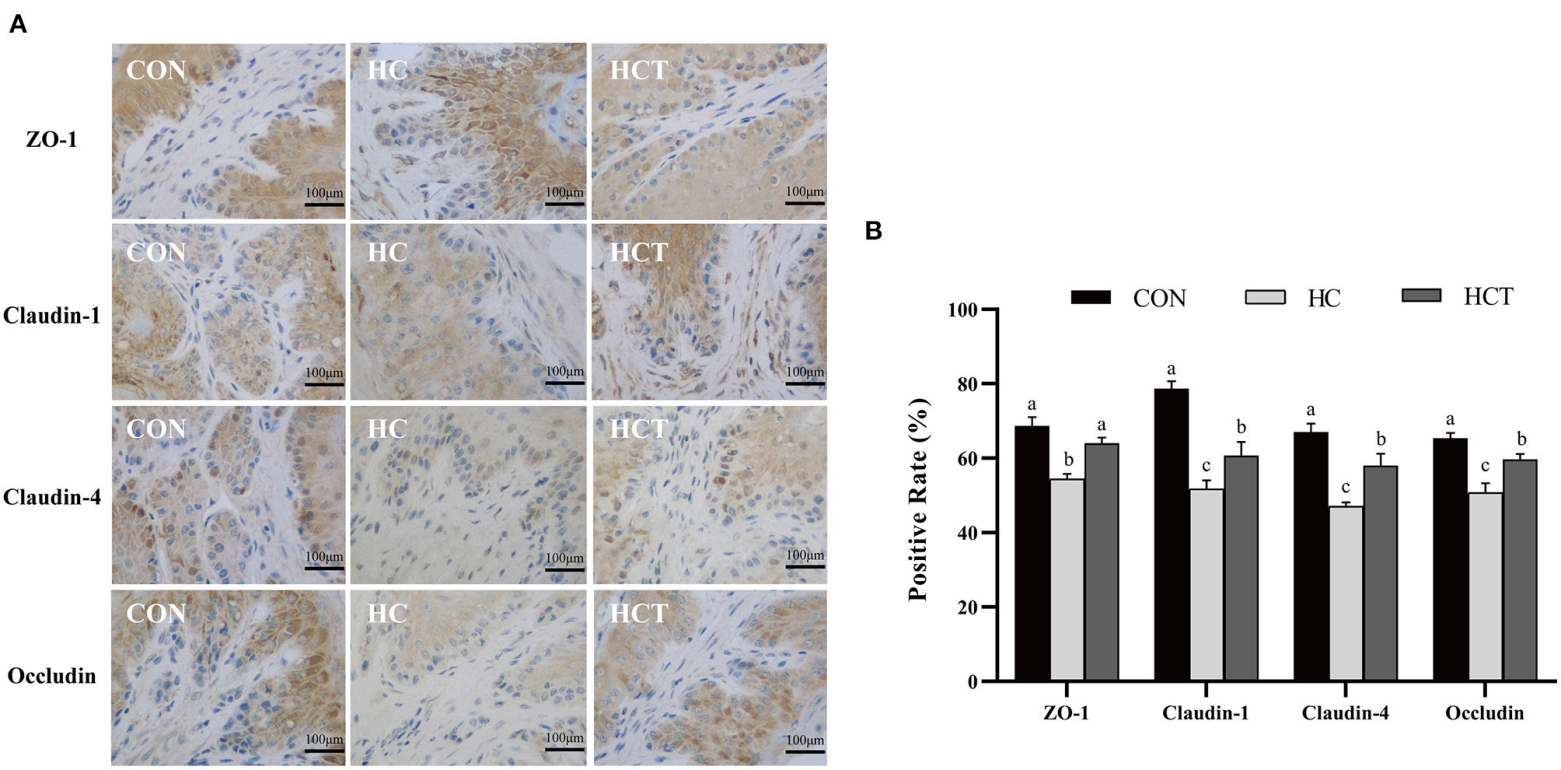
Effects of dietary thiamine supplementation on the expression and distribution of tight junction proteins during HC diet feeding. **(A)** Immunohistochemistry analysis of ZO-1, claudin-1, claudin-4, and occludin in the ruminal epithelium. **(B)** Positive rate analysis. All scale bars represent 100 μm (*n* = 8 goats/treatment). CON, low-concentrate diet; HC, high-concentrate diet; HCT, high-concentrate diet supplemented with 200 mg of thiamine/kg of dry matter intake. The mean values in columns without a common superscript letter differ (*P* < 0.05).

To investigate the differences in ruminal epithelium physical barrier with thiamine supplementation, we examined the expression and distribution of TJs (ZO-1, occludin, claudin-1, and claudin-4). The immunohistochemical analysis results showed that the protein expression levels of ZO-1, occludin, claudin-1, and claudin-4 were significantly lower in the HC diet compared with the CON treatment. The expression levels of ZO-1, occludin, claudin-1, and claudin-4 were also higher (*p* < 0.05; Figure 5) in the HCT diet than in the HC.

### Gene Expression

**Figure 6:**
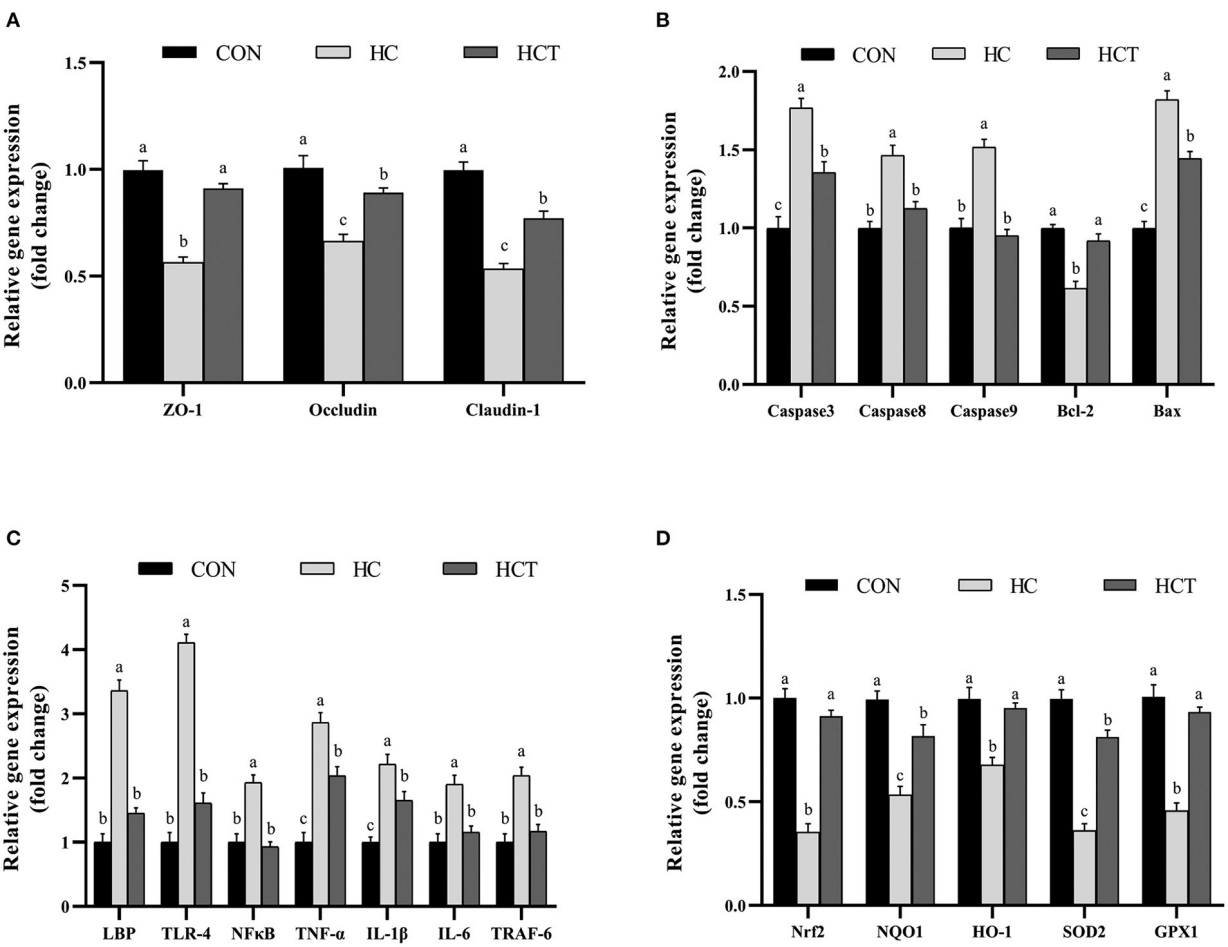
Effects of dietary thiamine supplementation on the mRNA abundance of genes during HC diet feeding. **(A)** Expression abundance of the tight junction-related gene. **(B)** Expression abundance of apoptosis-related genes. **(C)** Expression abundance of immune-related genes. **(D)** Expression abundance of oxidation-related genes (*n* = 8 goats/treatment). CON, low-concentrate diet; HC, high-concentrate diet; HCT, high-concentrate diet supplemented with 200 mg of thiamine/kg of dry matter intake. The mean values in columns without a common superscript letter differ (*P* < 0.05).

The mRNA expression of ZO-1, occludin, claudin-1, Bcl-2, Nrf2, SOD2, GPX1, NQO1, and HO-1 in the HCT treatment significantly increased in comparison with the HC diet treatment (*p* < 0.05; Figure 6). Meanwhile, the mRNA expression of caspase 3, caspase 8, caspase 9, Bax, LBP, TLR-4, NFκB, TNF-α, IL-1β, IL-6, and TNF receptor-associated factor six had a significant decrease in the HCT treatment in comparison with the HC diet treatment (*p* < 0.05; Figure 6).

### Protein Expression

**Figure 7:**
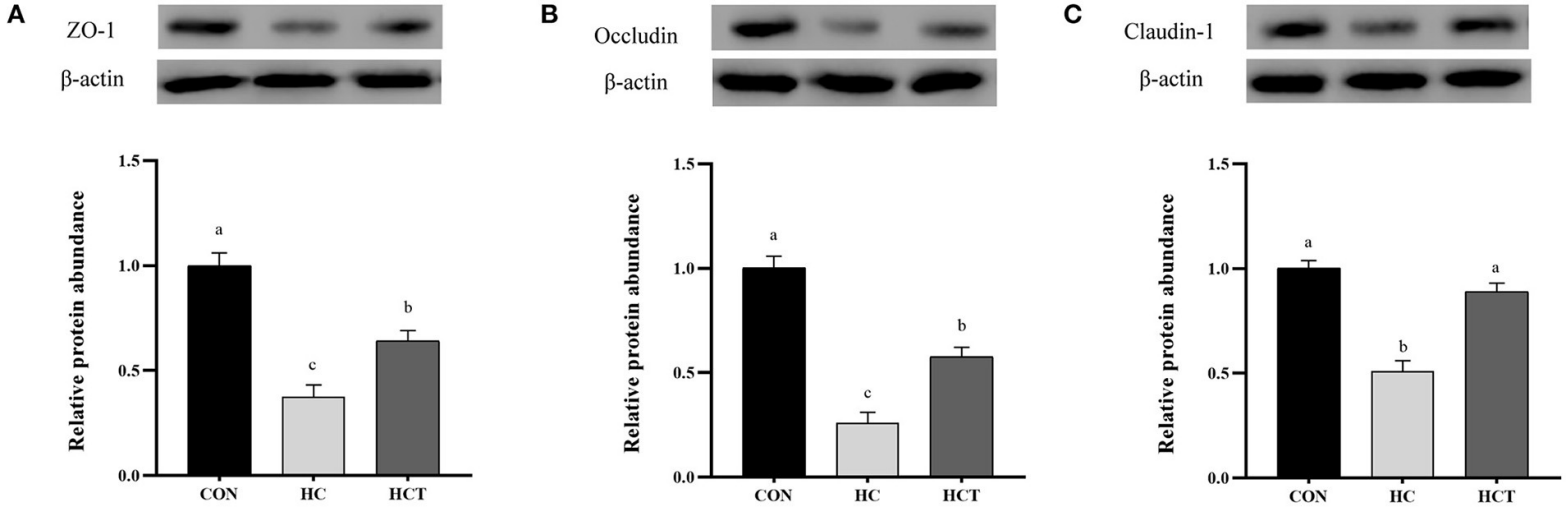
Effects of thiamine supplementation on the tight junction relative protein **(A)** ZO-1, **(B)** Occludin and **(C)** Claudin-1 expression levels in the ruminal epithelium of goats during the HC diet feeding (*n* = 8 goats/treatment). CON, low-concentrate diet; HC, high-concentrate diet; HCT, high-concentrate diet supplemented with 200 mg of thiamine/kg of dry matter intake. The mean values in columns without a common superscript letter differ (*P* < 0.05).

**Figure 8:**
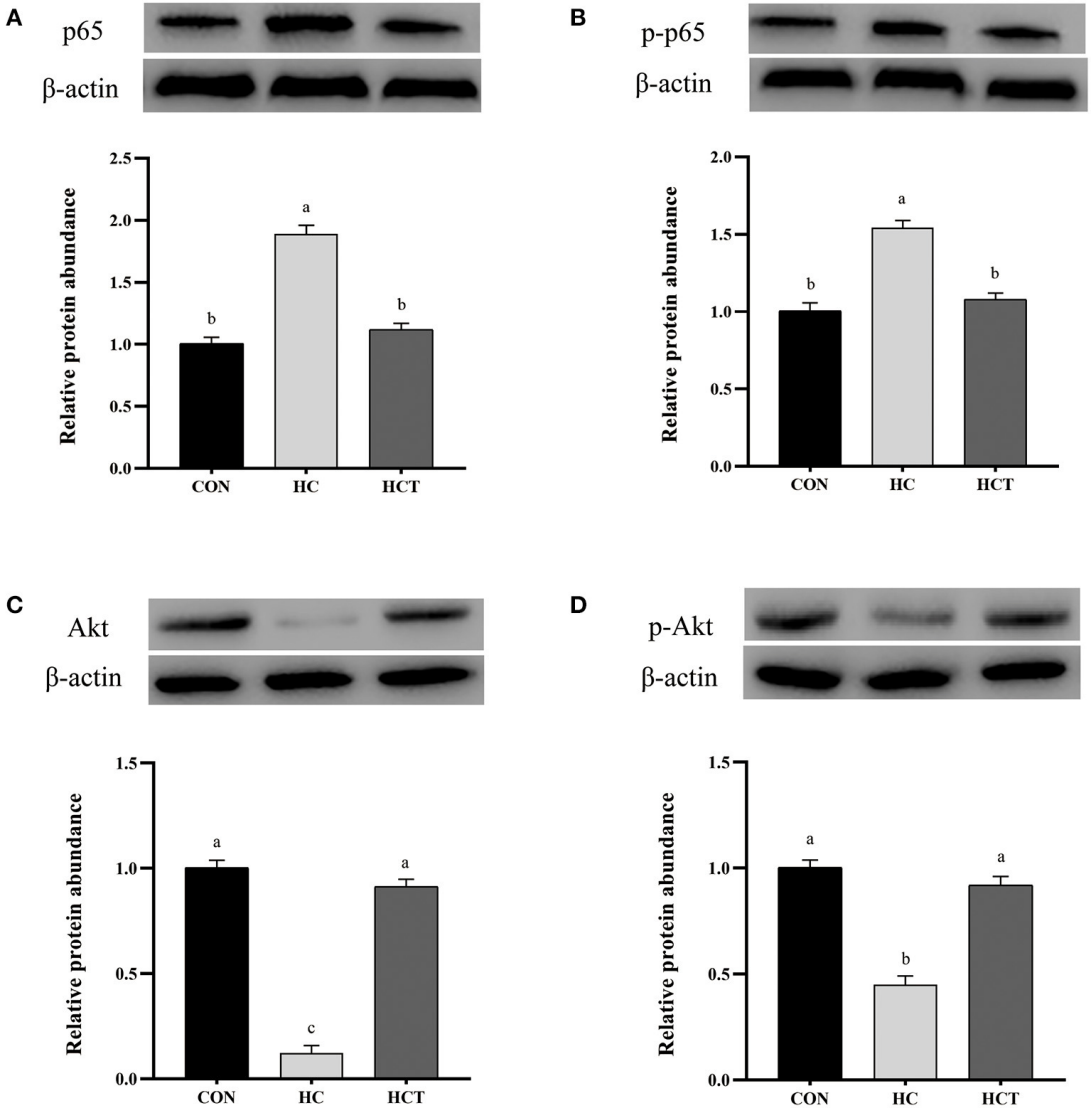
Effects of thiamine supplementation on the immune relative protein **(A)** p65, **(B)** p-p65, **(C)** Akt, and **(D)** p-Akt expression levels in the ruminal epithelium of goats during the HC diet feeding (*n* = 8 goats/treatment). CON, low-concentrate diet; HC, high-concentrate diet; HCT, high-concentrate diet supplemented with 200 mg of thiamine/kg of dry matter intake. The mean values in columns without a common superscript letter differ (*P* < 0.05).

**Figure 9:**
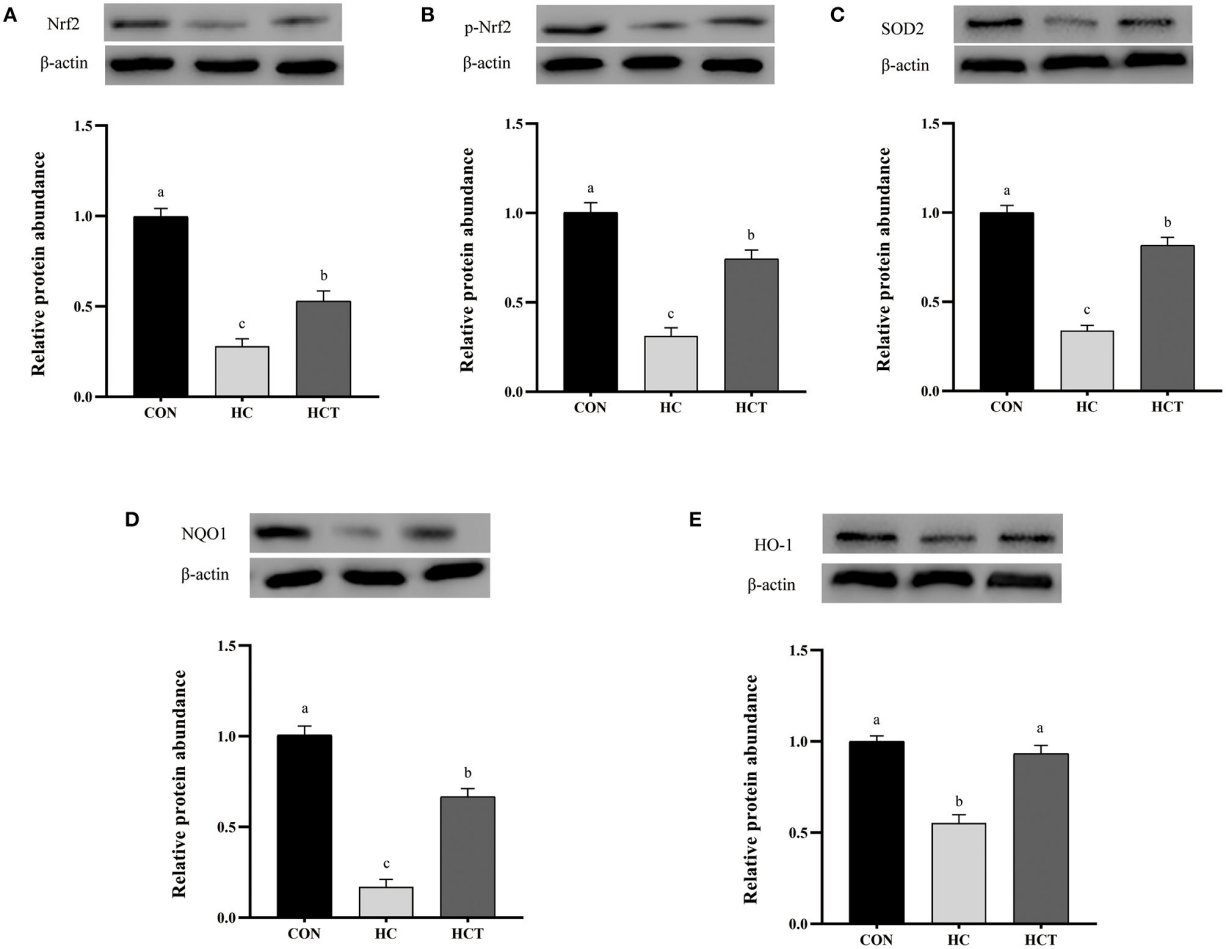
Effects of thiamine supplementation on the oxidative relative protein **(A)** Nrf2, **(B)** p-Nrf2, **(C)** SOD2, **(D)** NQO1 and **(E)** HO-1 expression levels in the ruminal epithelium of goats during the HC diet feeding (*n* = 8 goats/treatment). CON, low-concentrate diet; HC, high-concentrate diet; HCT, high-concentrate diet supplemented with 200 mg of thiamine/kg of dry matter intake. The mean values in columns without a common superscript letter differ (*P* < 0.05).

Compared with the CON, the HC diet significantly reduced the protein expression of ZO-1, occludin, claudin-1, NQO1, HO-1, SOD2, Akt, p-Akt, Nrf2, and p-Nrf2; conversely, the expression of NFκB-related proteins p65 and pp65 was significantly increased (*p* < 0.05; Figures 7–9). Compared with the HC treatment, dietary thiamine supplementation reversed this change for those indicators (*p* < 0.05).

## Discussion

Although the excessive amounts of high-concentrate diet in ruminants can enhance growth and milk yield for the short term, the accumulation of organic acids and plummeting of ruminal pH generate translocation of LPS, resulting in the damage of the rumen epithelium barrier (40, 41). In the current study, the HC diet feeding for 12 weeks exhibited a low pH and high VFA content compared to the CON treatment, which are characteristics of SARA. We found that the HCT diet induced a higher ruminal pH than that in the HC diet and reversed the negative change of VFA and LPS. The results indicated that thiamine supplementation changed the rumen fermentation state, which could be partially attributed to the evolution in microbial composition. Thiamine is indispensable for the proliferation of some ruminal bacteria strains of *Ruminococcus*, such as *Ruminococcus albus* and *Ruminococcus flavefasciens* (42). Meanwhile, relevant studies also uncovered that greater ruminal thiamine concentration could facilitate the increase in pH, which helped the growth of *Succinivibrio* (43). These results may provide more indirect clues for us to study the protective effect of thiamine on the epithelial barrier.

Correlative research has demonstrated that ruminal barrier function in ruminants has a molecular adaptation mechanism for HC diet feeding in the short term (44), and no impairment can be detected in cell layers during a short exposure (did not exceed 21 days) to the HC diet (6, 45). Thus, we fed the goats with the HC diet (70% grain) lasting for 12 weeks to monitor the structure of the rumen epithelium. The histological analysis indicated that the HC diet with thiamine supplementation for a long-term feeding regime protects the ruminal barrier. Furthermore, the thiamine effectiveness evidence in ruminal barrier function protection was demonstrated by SEM of the papillae surface.

Epithelial tight junction proteins are highly dynamic structures that contribute to the physical barrier function and play an important role in maintaining cell–cell interactions and stabilizing the paracellular and transcellular pathways (46). Occludin contributes to the maintenance of the TJ barrier as the large-channel TJ pathway, the pathway responsible for the macromolecule flux (47), with ZO-1 as an organizing component of the tight junction link occludin to the cortical actin cytoskeleton (11). Therefore, the coordination of occludin and ZO-1 plays a crucial role in maintaining the epithelium barrier. Claudins consist of multiple gene families, which have the ability to regulate cell proliferation (48). Our immunohistochemistry results demonstrated that the HCT diet defended the rumen epithelial physical barrier function compared to the HC diet as evidenced by the significantly altered expression and distribution of rumen epithelial tight junction proteins (ZO-1, occludin, claudin-1, and claudin-4). The expression of TJ gene and protein was also in accordance with the results of immunohistochemistry. Recent studies showed that the HC diet increased the ruminal epithelial permeability in goats by affecting the expression of tight junction proteins (36, 49). Thus, the Ussing chamber trial was chosen in this study for analysis of permeability and electrophysiology with thiamine supplementation.

Electrophysiology is associated with a balanced ruminal concentration of total VFA. Klevenhusen et al. indicated that a drop in pH causes an increase in the capacity of Isc (8). An excess of butyrate is toxic and can cause hyperkeratosis (50). Our study demonstrates that the HC diet increased Isc and Gt as well as decreased PD of the ruminal epithelium of goats and increased the permeability through the ruminal wall of the marker molecules FITC and HRP. Paracellular permeability can be estimated by the transepithelial transport of molecules, which usually are not present in the extracellular domain. Therefore, the value rise in Isc and PD, coupled with lower permeability to FITC and HRP, indicates an increased epithelial barrier function with thiamine supplementation. Thiamine supplementation changed the components of VFA as described above, which could be ascribed to the improvement in microbial community.

Apoptosis and damage of rumen epithelium are simultaneous during long-term high-concentrate diet feeding (51). Apoptosis plays a vital role in tissue homeostasis and development. Nevertheless, excessive apoptosis harms the barrier equilibrium of epithelium (52). Apoptosis may be triggered through the death receptor pathway, and the intrinsic apoptosis pathway depends on the mitochondria (53). In the present study, the HCT diet downregulated the proapoptotic genes such as Bax, caspase 3, caspase 8, and caspase 9 but upregulated the antiapoptotic gene Bcl2. The expression of Fas tends to decline, although the change was not statistically significant, which indicates a close relationship between apoptosis mediated by both the Fas and mitochondria. The change in the expression of caspase family members genes indicated a decrease in apoptosis rate with thiamine. The result of TUNEL analysis is also evidence that supports the change of gene expression. It has been reported that oxidative stress has great effects on mediating apoptosis (54). The ability of oxidative stress to trigger apoptosis as a result of a large number of cellular damages has been related to lipid peroxidation and alterations in proteins and nuclei (55). The fact has been revealed that thiamine deficiency caused an upregulation of apoptosis, inducing factor gene expression and leading to caspase 3-mediated apoptosis (56), while supplementation of thiamine ameliorated apoptosis by increasing the levels of GPX, SOD, and GSH (57). All these shreds of evidence point to the link between epithelial barrier function and oxidative stress with thiamine supplementation.

Our results have suggested that feeding a high-concentrate diet to goats for a long period results in the accumulation of LPS and VFA in the rumen. Previous studies have uncovered that suppression of the antioxidant system and activation of the immune system were caused by the high-concentrate diet through an accumulated LPS concentration in peripheral blood (4, 28), which is consistent with our findings for the expression of inflammatory and oxidative genes. We have previously reported that thiamine activates the immune system during high-concentrate feeding (4, 32). The increase of inflammatory factors in plasma indicates the occurrence of inflammation; regulation of IL-6 release by IgA may be among the anti-inflammatory mechanisms preventing the uncontrolled release of potentially noxious levels of inflammatory cytokines during acute or chronic responses (58). The NFkB pathway is known to regulate the expression of TJ proteins. It has been shown that decreases of TJ proteins produced chronic inflammation, in which NFkB activation plays a critical role (59).

Although oxidative reactions are crucial for animals, an excess of this reaction may cause tissue damage, while the occurrences of ROS exceeding the capacity of antioxidant defenses may cause oxidative stress (60). In the current study, we observed that MDA activity in the HCT treatment was significantly lower and that GSH, GSH-PX, SOD, and T-AOC were significantly higher than those in the HC treatment. These responses were similar for these indicators in epithelial tissue. Abaker et al. (61) demonstrated that a high-concentrate diet caused low levels of GPX and T-AOC and high levels of MDA in plasma. Watson et al. (26) proved the decreased SOD and GPX activities in male rats following endotoxin administration. Sokołowska et al. (62) also discovered that LPS induced oxidative stress and inflammatory reaction in the rat striatum. These studies give us an implication that LPS may induce oxidative stress through increasing of LPS in a high-concentrate feeding period. Previous research has shown that oxidative damage occurs along with oxidative stress caused by an increased level of MDA in serum (63). Multifarious antioxidant enzymes like SOD and GSH-PX play a vitally important role in protecting organisms from oxidative damage (64). The deficiency of thiamine may significantly impair the activity of transketolase, which is indispensable for the maintenance of GSH activity, a crucial antioxidant and free radical scavenger. Meanwhile, a previous study also showed that thiamine normalizes lipid peroxidation levels and elevates glutathione reductase activity (65).

Nrf2 acts as a sensor for oxidative stress, which regulates antioxidant defense systems (66). Nrf2 is restrained by Kelch-like ECH-associated protein 1 (keap1) in the cytoplasm under a situation of normal physiology; keap1 inhibits the function of Nrf2 by retaining Nrf2 in the cytoplasm under normal physiological situations. Nrf2 forms heterodimers with small Maf proteins and binds to the antioxidant response elements of target genes including GSH-Px, CAT, and SOD together with NQO1 and HO-1 (51) when cells are exposed in stress conditions (67). As a gene of anti-inflammatory and antioxidant properties, HO-1 is an Nrf2 target gene in the public eye (68). NQO1 can cope with oxidative lesions by weakening the activity of NADPH oxidase as well as the secretion of ROS, which is an Nrf2-mediated phase II metabolizing enzyme (69). The redox-regulated ubiquitous transcription factor NFκB can be activated by oxidative stress and inhibited by various antioxidants (23). The equilibrium state of both Nrf2 and NFκB regulation is vitally important to maintain the redox homeostasis in the health and performance of the body (29). Chen et al. revealed that Nrf2 deficiency impairs barrier function by disrupting the integrity of energy-dependent TJ (70), while the Nrf2 activator, quercetin, significantly enhanced intestinal barrier function through upregulation of claudin-4 in Caco-2 cells (71). Previous research has also shown that NFκB can damage barrier function through motivating the release of inflammatory factors (72).

The NFκB and Nrf2 pathways interface at several points to regulate transcription or the function of their downstream targets. On one hand, Nrf2 and NFκB can be functionally antagonistic. The down-regulated expression of Nrf2 induces more intense inflammation through activation of NFκB and down-stream proinflammatory cytokines (73). On the other hand, NFκB and Nrf2 both regulate a subset of target genes such as HO1 and IL-8. In the present study, thiamine increased the Nrf2 protein level and up-regulated the levels of antioxidant enzymes such as SOD2 together with phase II metabolizing enzymes (like NQO1 as well as HO-1), all of which were conducive to relieve oxidative lesions in the ruminal epithelium for high-concentrate diet feeding. At the same time, our research also showed the decline of NFκB-associated proteins p65 and pp65 as well as the increase of Akt and pAkt during thiamine supplement. Some studies also point out that NFκB-p65 and Nrf2 had synergy in regulating the antioxidative response in these cells (74). Moreover, the PI3K/AKT pathway is involved in the induction of Nrf2-driven gene expression (75). These studies all point to one piece of evidence that thiamine reduces oxidative stress by regulating the Nrf2–NFκB pathways during a high-concentrate feeding.

In conclusion, the current study results revealed that the rumen epithelial barrier function and the immune function were impaired, which are associated with the apoptotic and oxidative status that occurred during 12 weeks of feeding with the high-concentrate diet. Dietary thiamine supplementation was able to alleviate those harmful effects through the NFκB and Nrf2 signaling pathways. Thus, these findings provide that thiamine could potentially serve as a dietary supplementation to help in the alleviation of the negative effects of SARA in high-grain, intensive ruminant production.

## Data Availability Statement

The raw data supporting the conclusions of this article will be made available by the authors, without undue reservation.

## Ethics Statement

The animal study was reviewed and approved by Yangzhou city.

## Author Contributions

YM and HW designed the research. YM and HZ conducted the research. YM and YZ analyzed the data. YM wrote the paper and had primary responsibility for the final content. ME revised the language of the current paper and carried out the experiment. All authors read and approved the final manuscript.

## Conflict of Interest

The authors declare that the research was conducted in the absence of any commercial or financial relationships that could be construed as a potential conflict of interest.
