# Erratum: Thiamine Alleviates High-Concentrate-Diet-Induced Oxidative Stress, Apoptosis, and Protects the Rumen Epithelial Barrier Function in Goats

**DOI:** 10.3389/fvets.2021.715703

**Published:** 2021-06-25

**Authors:** Frontiers Production Office

**Affiliations:** Frontiers Media SA, Lausanne, Switzerland

**Keywords:** thiamine, goats, subacute rumen acidosis, apoptosis, oxidative stress, immune function, tight junction proteins

Due to a production error, there was a mistake in the author list. Author Mawda Elmhadi was omitted from the author list as the third author.

The publisher apologizes for this mistake. The original version of this article has been updated.

