# Corrigendum: Thiamine Alleviates High-Concentrate-Diet-Induced Oxidative Stress, Apoptosis, and Protects the Rumen Epithelial Barrier Function in Goats

**DOI:** 10.3389/fvets.2022.859615

**Published:** 2022-02-23

**Authors:** Yi Ma, Ying Zhang, Mawda Elmhadi, Hao Zhang, Hongrong Wang

**Affiliations:** Laboratory of Metabolic Manipulation of Herbivorous Animal Nutrition, College of Animal Science and Technology, Yangzhou University, Yangzhou, China

**Keywords:** thiamine, goats, subacute rumen acidosis, apoptosis, oxidative stress, immune function, tight junction proteins

In the original article, there was a mistake in Figure 4 and Figure 5, the use of scale bar were not standardized. The corrected Figure 4 and Figure 5 appear below.

**Figure 4:**
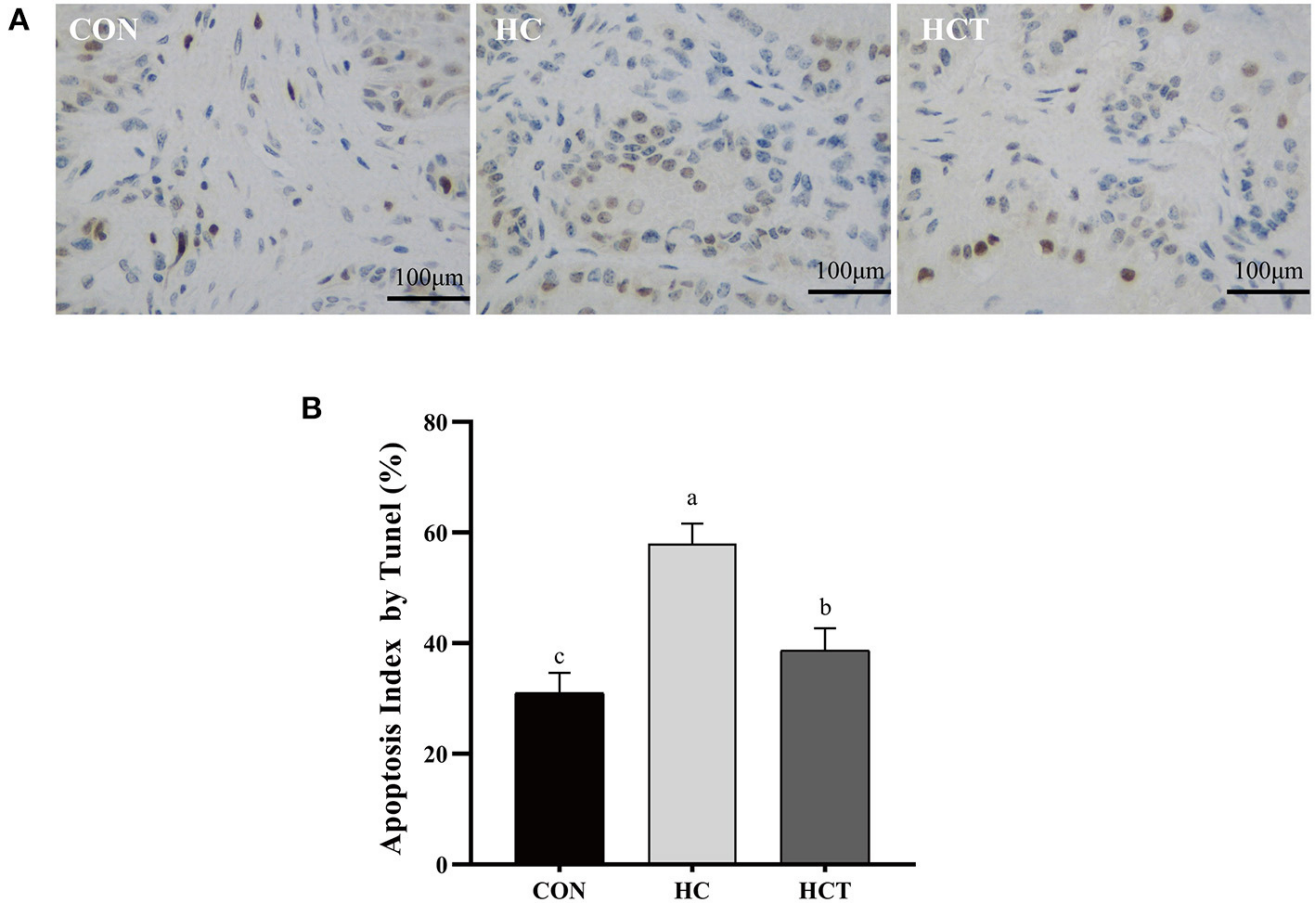
TUNEL comparison of the ruminal epithelium from the low-concentrate diet (CON), high-concentrate diet (HC), and high-concentrate diet with thiamine (HCT) treatments. Ruminal epithelium samples from each treatment were processed for evaluation of TUNEL-positive apoptotic cells. **(A)** Representative sections of rumen from the different treatments (CON, HC, and HCT treatments). **(B)** Apoptosis index analysis. All scale bars represent 25 μm (*n* = 8 goats/treatment). The mean values in columns without a common superscript letter differ (*P* < 0.05).

**Figure 5:**
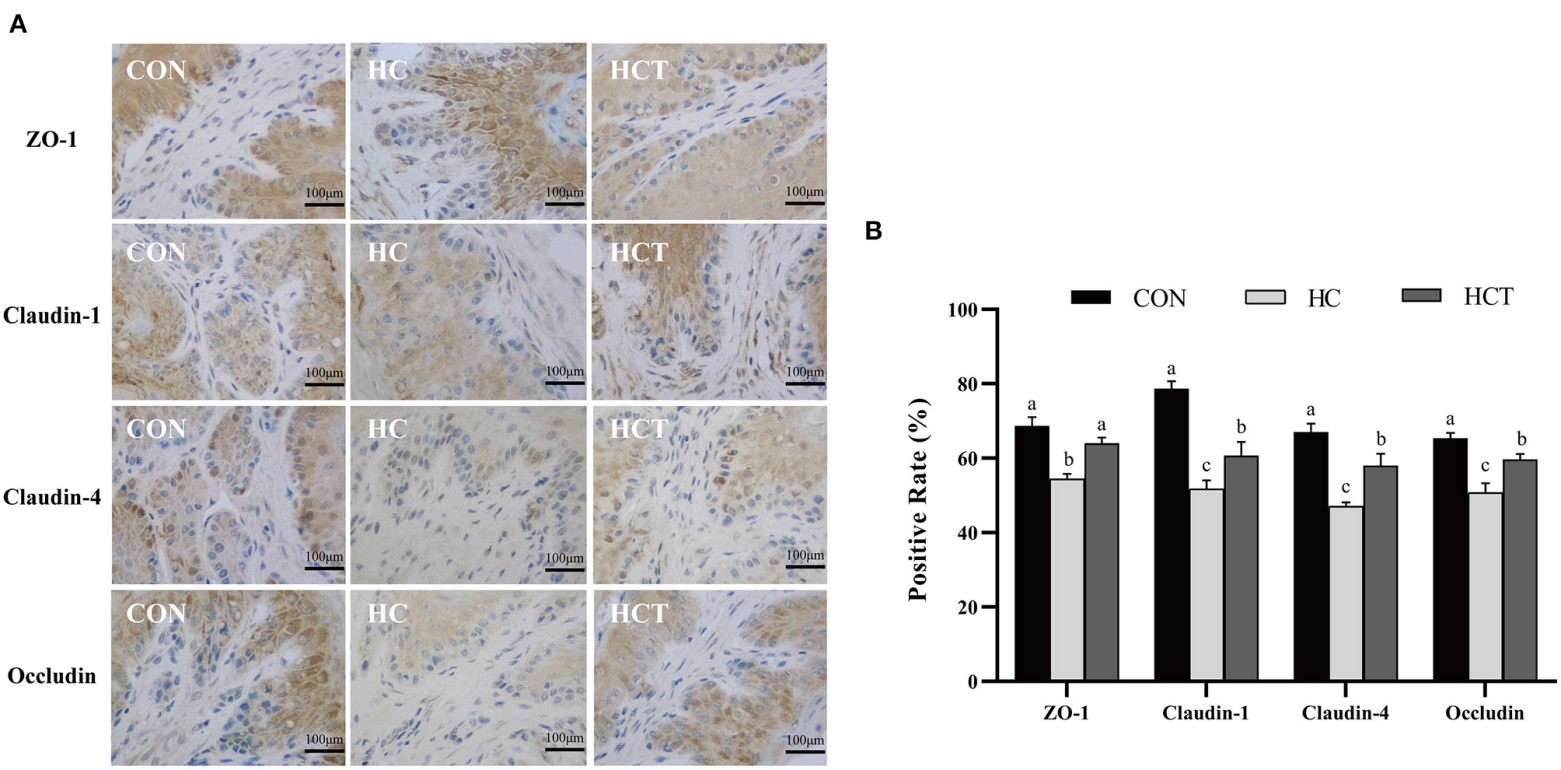
Effects of dietary thiamine supplementation on the expression and distribution of tight junction proteins during HC diet feeding. **(A)** Immunohistochemistry analysis of ZO-1, claudin-1, claudin-4, and occludin in the ruminal epithelium. **(B)** Positive rate analysis. All scale bars represent 100 μm (*n* = 8 goats/treatment). CON, low-concentrate diet; HC, high-concentrate diet; HCT, high-concentrate diet supplemented with 200 mg of thiamine/kg of dry matter intake. The mean values in columns without a common superscript letter differ (*P* < 0.05).

Mawda Elmhadi's contributions were not listed in the original article. The corrected **Author Contributions** statement appears below.

## Author Contributions

YM and HW designed the research. YM and HZ conducted the research. YM and YZ analyzed the data. YM wrote the paper and had primary responsibility for the final content. ME revised the language of the current paper and carried out the experiment. All authors read and approved the final manuscript.

The authors apologize for these errors and state that this does not change the scientific conclusions of the article in any way. The original article has been updated.

## Publisher's Note

All claims expressed in this article are solely those of the authors and do not necessarily represent those of their affiliated organizations, or those of the publisher, the editors and the reviewers. Any product that may be evaluated in this article, or claim that may be made by its manufacturer, is not guaranteed or endorsed by the publisher.

